# A Deep Recurrent Learning-Based Region-Focused Feature Detection for Enhanced Target Detection in Multi-Object Media

**DOI:** 10.3390/s23177556

**Published:** 2023-08-31

**Authors:** Jinming Wang, Ahmed Alshahir, Ghulam Abbas, Khaled Kaaniche, Mohammed Albekairi, Shahr Alshahr, Waleed Aljarallah, Anis Sahbani, Grzegorz Nowakowski, Marek Sieja

**Affiliations:** 1 College of Information Science & Technology, Zhejiang Shuren University, Hangzhou 310015, China; 2 Department of Electrical Engineering, College of Engineering, Jouf University, Sakakah 72388, Saudi Arabia; 3 School of Electrical Engineering, Southeast University, Nanjing 210096, China; 4 Institute for Intelligent Systems and Robotics (ISIR), CNRS, Sorbonne University, 75006 Paris, France; 5 Faculty of Electrical and Computer Engineering, Cracow University of Technology, Warszawska 24 Str., 31-155 Cracow, Poland

**Keywords:** image analysis, feature extraction, region of interest, hotspot, target detection, multi-object, deep reinforcement learning (DRL)

## Abstract

Target detection in high-contrast, multi-object images and movies is challenging. This difficulty results from different areas and objects/people having varying pixel distributions, contrast, and intensity properties. This work introduces a new region-focused feature detection (RFD) method to tackle this problem and improve target detection accuracy. The RFD method divides the input image into several smaller ones so that as much of the image as possible is processed. Each of these zones has its own contrast and intensity attributes computed. Deep recurrent learning is then used to iteratively extract these features using a similarity measure from training inputs corresponding to various regions. The target can be located by combining features from many locations that overlap. The recognized target is compared to the inputs used during training, with the help of contrast and intensity attributes, to increase accuracy. The feature distribution across regions is also used for repeated training of the learning paradigm. This method efficiently lowers false rates during region selection and pattern matching with numerous extraction instances. Therefore, the suggested method provides greater accuracy by singling out distinct regions and filtering out misleading rate-generating features. The accuracy, similarity index, false rate, extraction ratio, processing time, and others are used to assess the effectiveness of the proposed approach. The proposed RFD improves the similarity index by 10.69%, extraction ratio by 9.04%, and precision by 13.27%. The false rate and processing time are reduced by 7.78% and 9.19%, respectively.

## 1. Introduction

Object detection from populated images is a complicated task in an application or system. Object detection is mostly used in computer vision and image-processing systems. Populated images contain various factors and things presented among people [1]. Object detection from populated images consumes more time when compared with normal images. Deep learning-based techniques are widely used in the object detection process. Deep learning is used to implement the feature extraction approach, which pulls out the key patterns and features in the image [2]. The extracted features produce necessary information to object detection that reduces latency in computation and classification processes. Some of the important aspects and details are extracted by deep learning that produces optimal data for further processes in the detection method [3]. The many items and things in populated images decrease the system’s energy efficiency range. The convolutional neural network (CNN) algorithm minimizes computing time and energy requirements, enhancing system performance and effectiveness [4]. CNN detects the actual data which are presented in populated images. CNN maximizes accuracy in object detection, which enhances the effectiveness of detection and prediction processes [5].

The ideal information needed for the object detection procedure is provided by region of interest (ROI) detection. Information that is essential to the detecting process is contained in populated images. In the classification and segmentation process, ROI lowers latency. ROI gives pertinent data necessary for the object detection procedure [6]. The ROI-based object detection method mostly uses CNN and artificial intelligence (AI) techniques to enhance the energy-efficiency range of the systems. Both local and global regions are detected from populated images [7]. ROI increases the system’s viability, robustness, and efficiency levels by maximizing the total accuracy of the object detection process. CNN-based models are mostly used for the ROI detection process, predicting spatial regions and important pixels from an image [8]. The pack and detect (PaD) method is also used in ROI detection. The PaD method reduces the requirements required for the object detection process. The requirements are reduced based on ROI, which produces optimal information for object detection. Both spatial and temporal regions are detected from the given image, which reduces the complexity of the object classification process [9,10].

Object detection uses machine learning (ML) methods. The primary goal of ML approaches is to increase the object detection process’s accuracy range. ML-based techniques are commonly used to identify the important region of interest (ROI) from populated images [11]. The deep reinforcement learning (DRL) algorithm identifies the ROI in object detection. DRL achieves high ROI detection accuracy, reducing latency in the classification and optimization process [12]. DRL employs a feature extraction technique to extract pertinent data from an image. The performance and effectiveness of the object detection process are enhanced by DRL. To detect objects, the deep neural network (DNN) technique is also employed [13]. The ROI provides the DNN with the information it needs, decreasing the time and energy needed for computation and classification. The DNN lowers the computation’s level of complexity, increasing the systems’ relevance and viability. Object detection is used in DNN, which detects the data required for object detection [14]. The DNN improves object detection’s accuracy range, which lowers mistakes in subsequent detection and prediction procedures. The support vector machine (SVM) technique, which extracts accurate data from ROI datasets, is frequently employed in object detection. SVM performs an image classification process that identifies the objects presented in an image [15].

The main contribution of the paper is

- Designing the region-focused feature detection (RFD) method to improve target detection accuracy.
- Evaluating the deep recurrent learning mathematical model to iteratively extract these features using a similarity measure from training inputs corresponding to various regions.
- According to the experimental outcomes, the suggested RFD model enhances the similarity index, extraction ratio, precision, and false rate and reduces processing time.

The rest of the article is arranged as follows: Section 2 deliberates related works, Section 3 proposes the RFD framework, and Section 4 executes the experiments and shows the results. In Section 5, a discussion has been provided. Finally, Section 6 concludes the research paper.

## 2. Related Works

Kim et al. [16] proposed a bounding-box critic network (BBC Net) framework for object detection. BBC Net identifies the exact occlusions and object regions presented in an image. Based on specific traits and patterns, object categories and parameters are categorized. BBC estimates the crucial areas and pixels from images to reduce computing latency. The suggested BBC Net framework successfully detects objects with high accuracy.

A context-driven detection network (CDD-Net) for multiple-class object detection was introduced by Wu et al. [17]. The newly developed framework mainly uses remote-sensing images, which give the detection process the data and patterns it needs. In this study, features and nearby objects from an image are detected using a local context feature network (LCFN). According to experimental findings, the suggested CDD-Net architecture improves object detection accuracy, expanding the performance envelope of the systems.

A hierarchical context embedding (HCE) framework for region-based item detection was created by Chen et al. [18]. In this study, precise patterns and regions of objects are detected from an image using region-based detectors. The HCE framework also detects region of interest (ROI) features and parameters, which lowers the detection process’s time and energy requirements. The suggested HCE architecture raises the systems’ overall efficacy and viability by enhancing object detection accuracy.

A patch-based three-stage aggregation network (PTAN) was created by Sui et al. [19] for object detection. The main application of the suggested system is object detection in high-resolution remote-sensing images. PTAN maximizes the quality of the ROI and features that provide optimal information for further detection. A patch-based strategy is mostly used here to train the parameters and regions presented in an image. Compared with other frameworks, the proposed PTAN framework achieves high accuracy in object detection for remote-sensing images.

Chen et al. [20] introduced a deep neural network method named RoIFusion for the 3D object detection process. RoIFusion merges the multi-modality features and patterns to identify the exact objects required from the image. ROI and pixel detection levels are reduced by the RoIFusion method, which reduces the energy consumption range in computation. The systems’ effectiveness and dependability range is increased by the suggested RoIFusion approach.

Han et al. [21] designed a compressive sensing (CS)-based atomic force microscopy (CS-AFM) scheme for object detection systems. Both high- and low-resolution images are used in CS-AFM that detects accuracy pixels for further processes. Scanners and detectors are used here to predict the objects’ class from an image. Supplementary scanning is implemented to finalize the types of objects. The proposed CS-AFM method reduces computation time, improving object detection’s quality and accuracy range.

A cascaded multi-D-view fusion approach (CM3DV) for object detection was created by Sun et al. [22]. This work uses a cascaded multi-view feature fusion module to determine the specific categories of items from 3D images. Modulated rotation head (MRH) is developed by the CM3DV model, providing objects’ necessary features and patterns. According to experimental data, the suggested CM3DV approach maximizes the object detection process’s overall precision and energy efficiency levels.

An encoder-steered multi-modality feature guidance network (EFGNet) for RGB image and depth map salient item recognition was introduced by Xia et al. [23]. It is possible to extract from RGB images the unimodal characteristics and patterns required for object detection. Unimodal features deliver precise information about objects. The newly developed EFGNet approach improves the systems’ efficiency by improving object detection accuracy.

A multi-level fusion detection (MFD) algorithm-based object detection technique was created by Peng et al. [24]. Here, essential features and patterns present in heterogeneous images are extracted via feature extraction. The collected features give the object detection procedure the best data possible. Additionally, MFD recognizes the pixel and vision range from an image, reducing computation latency. The created MFD increases the viability and performance of object-detecting systems.

Yue et al. [25] developed a low-light image salient object detection (LLISOD) network for various applications. An unfolded implicit non-linear mapping (UINM) module is used here that detects the features for polishing feature maps. Object detection in the application from the low-light image is challenging. The suggested LLISOD framework lowers the detection process’s energy and time requirements. According to experimental data, the suggested LLISOD framework achieves good object detection accuracy.

Xu et al. [26] proposed a two-stage 3D object detection method using position encoding. Position encoding produces information useful for the detection process from raw point data and aggregating voxel features. Here, the major purpose of the feature aggregation module is to lower the error and latency range in the object detection process. From provided 3D images, context and specifics are derived. The proposed method improves object detection accuracy when compared to previous methods.

A corners-based fully convolutional network (C-FCN) for visual object identification systems was introduced by Jiao et al. [27]. This work predicts objects in an image’s right and left corners using a corner region proposal network (CRPN). The FCN is mainly used here to identify the end-to-end objects presented in an image. The FCN increases the accuracy of object detection with a minimum energy consumption range. Object detection is made simpler and more effective by the newly developed C-FCN approach.

A deep-wise separable convolutional network (D-SCNet) was created by Quan et al. [28] for object detection. This application uses the region convolutional neural network (R-CNN) technique to recognize an image’s key details and elements. In this work, a feature map is employed to offer the features and patterns of an object that are essential to the detection process. The suggested D-SCNet method improves the object identification process’s mobility, feasibility, stability, and accuracy compared to previous methods.

A framework for object detection based on graph neural networks was suggested by You et al. [29]. The primary goal of the suggested framework is to establish a connection between an image’s label embedding space and visual feature space. Both region and label proposals are detected from the relation graph, reducing the time consumption level in object classification. The proposed method is mainly used to perform relational reasoning in object detection. The proposed strategy broadens the scope of the reasoning process’s applicability and efficacy.

In their study, Pathak et al. [30] introduce an innovative approach for detecting faults in photovoltaic panels. This method involves analyzing thermal images of solar panels, which are obtained using a thermographic camera. Two advanced convolutional neural network models are employed in this study. The primary objective of the first model is to accurately classify the type of fault affecting the panel. Meanwhile, the second model is specifically designed to identify the region of interest within the faulty panel. The proposed approach employs the F1 score as a metric for evaluating and comparing multiple classification models. Among these models, the ResNet-50 transfer learning model achieves the highest F1 score.

A cross-diffusion-based salient object recognition approach for compact images was introduced by Wang et al. [31]. In this case, the cross-diffusion technique extracts the key details and areas from an image. The salient object detection procedure receives the best information possible from the retrieved features. The error range in detection is reduced by extracting both high-level and low-level image features. The newly developed technique boosts object detection accuracy, enhancing system performance.

Choi and Kim [32] suggested a sensor fusion system that combines a thermal infrared camera with a LiDAR sensor to accurately detect and identify objects in low-visibility situations, such as at night or during the day. The system’s effectiveness was tested using experiments. It remotely calibrates the thermal infrared camera and LiDAR sensor using a 3D calibration target. The suggested sensor system and fusion algorithm show their promise for autonomous vehicle perception technologies by demonstrating their capacity to detect and identify objects in difficult settings.

Zhang et al. [33] developed a vehicle object detection method named candidate region aggregation network (CRAN). The primary objective of the suggested approach is to enhance the detection process’s ability to aggregate data. The majority of optimization issues are resolved by CRAN, which lowers the levels of computational time and energy usage. The procedure of detecting objects in vehicles is more effective and accurate according to the suggested strategy.

Rahman et al. [34] demonstrated that RetinaNet (R101-FPN) and YOLOv5n could detect weeds in cotton fields, with RetinaNet having higher accuracy and YOLOv5n having the ability to be used in real time on devices with limited resources. The study emphasizes the value of data augmentation in enhancing weed identification model accuracy. Creating reliable and effective weed detection systems can be a key component of sustainable agriculture and weed management methods by utilizing deep learning capabilities and continual improvement in model training.

Dai and Nagahara [35] proposed a distributed safety control mechanism for multi-agent systems and applications for collision avoidance of mobile robotic networks. In the suggested method, each agent corrects its control input by resolving a distributed optimization problem to maximize the effectiveness of a predetermined cooperative control strategy while ensuring fulfillment of the safety constraint. The usefulness of the current methodology was proved through case studies examining issues with circular/elliptical vehicle accident avoidance.

Ramachandran Alagarsamy and Dhamodaran Muneeswaran [36] suggested the reptile search optimization algorithm with deep learning for multi-object detection and tracking (RSOADL-MODT). Position estimation, tracking, and action recognition are all parts of the RSOADL-MODT model shown here. The steps involved include “object detection”, “object classification”, and “object tracking”. The feature extraction process is enhanced in the first stage of the described RSOADL-MODT method using a path-augmented RetinaNet-based (PA-RetinaNet) object detection module. The RSOA is employed as a hyperparameter optimizer to enhance the network potential of the PA-RetinaNet approach. Finally, the classification capabilities of a quasi-recurrent neural network (QRNN) classifier are utilized. To evaluate the efficacy of the RSOADL-MODT algorithm’s object detection results, extensive experimental validation is conducted on the DanceTrack and MOT17 datasets. The simulation results validated the advantages of the RSOADL-MODT method over competing DL methods.

Hossein Adeli et al. [37] discussed the brain-inspired object-based attention network for multi-object recognition and visual reasoning. The authors demonstrate how the attention mechanism greatly enhances the precision with which substantially overlapping digits can be categorized. The model achieves near-perfect accuracy in a visual reasoning task that requires comparing two objects, and it significantly outperforms larger models in generalizing to unseen stimuli. This study highlights the usefulness of object-based attention systems that glimpse things in rapid succession.

## 3. The Proposed RFD Framework

**Figure 1:**
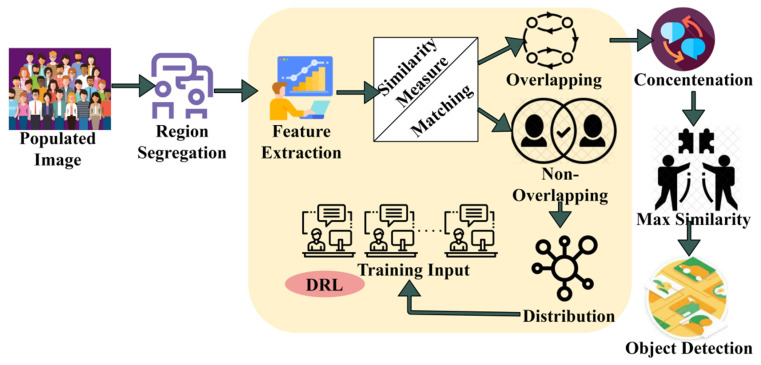
The Proposed RFD.

The issue of generic target detection is a crucial challenge in system vision. From the given densely populated image/video (scene), the target object detection is performed due to the presence of different objects. The proposed target object detection technology is designed to segregate the input image into the maximum possible regions to detect the precise target easily. In this generic target detection scenario, the feature types are considered first and then matched against targets in the raw imagery based on multiple objects, different locations, region of interest, scales, and orientations for exact target object detection. The features can be extracted to perform similarity measures from the training inputs’ feature matching in various regions through deep recurrent learning. Based on the similarity measure computation, the textural features are extracted with previously available information. This information matches the current features with existing ones for exploring generic target detection processes in the populated image. The proposed RFD technique performs precise target detection and textural feature extraction using a similarity measure in the maximum possible regions. The proposed RFD is illustrated in Figure 1.

A similarity measure is required, and a process from the training input target is provided for identifying overlapping and non-overlapping features in the original image. This matching process is performed to identify the target with maximum features due to multiple objects in the raw imagery at different locations. In non-overlapping feature identification, the region-wise feature distribution occurs for target detection. In distinguishable regions, the overlapping features are concatenated for detecting the exact target based on matching the target with training input using intensity and contrast features. The RFD technique functions between training input and a densely populated image. The overlapping and non-overlapping features are identified through similarity measures, where smart decisions and intelligent computations are made to identify the target. The smart decision for identifying overlapped and non-overlapped features is pursued using the training inputs from the given densely populated image $\left(P^{image}\right)$. The input raw imagery from any source S is processed, and the maximum possible regions can be segregated in which the contrast and intensity are evaluated. First, the two computational segments proposed are described to estimate the region selection and pattern matching. Features acquired for target detection include the type of object, edge, bounding box, color, textures, background information, position, and object labels.

### 3.1. Region Selection

**Figure 2:**
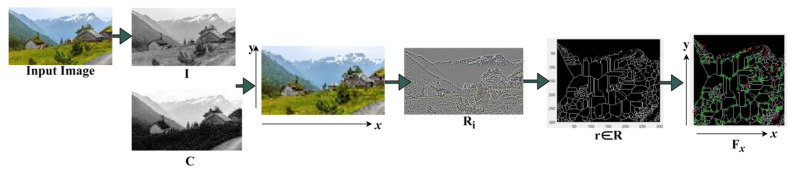
Region Segregation and Feature Extraction.

We compute region selection using intensity and contrast feature matching with the previously available data, and then we evaluate the basic region selection for each pattern. Meanwhile, in the raw imagery, features such as color, intensity, orientation, scale, and pixel distribution are employed. This proposed technique can extract multiple objects and new features to effectively identify the target. In this technique, the heterogeneous densely populated images/videos are analyzed along with multiple objects to perform feature extraction using deep recurrent learning iterations for computing similarity measures in different regions. The two features extracted in this paper are contrast and intensity. Note that the textural feature does not help since input images are often grayscaled. This study estimates the intensity and contrast features by analyzing 16×16 regions; each pixel is 16 times smaller than the raw imagery vertically and horizontally (1 pixel = 16×16 image region). The remaining features are extracted using a similarity measure or matching in different regions based on the training inputs. The given raw imagery is used for identifying the target based on the region selection process. The input populated image calculates the contrast and intensity features over different regions and objects/people. In this instance, the given image $P^{image}$ is estimated as in Equations (1)–(3):

(1) $${\left(P^{image}\right)}_{x,y}=RS\left(C,\;I\right)$$

(2) $$R_{i}=\frac{r\times \left(C_{Max}-C_{Min}\right)}{T}+I_{Min}$$

(3) $$Fx=\overset{T}{\underset{r}{\sum }}xy+ [\frac{\left(\frac{C_{Min}}{C_{Max}}-\frac{r}{R}\right)}{2\left(R-\bar{R}\right)}]$$

where RS means region selection based on intensity I and contrast C from the given $P^{image}$. The variables x and y denote the row and column of the image patch. Where r is the selected region for target object detection and r∈R, $\;C_{Max}$ and $C_{Min}$ represent the maximum and minimum contrast feature required in the input image at different processing time intervals T. The variables Fx and $\bar{R}$ are used to represent the feature extraction and previously available information for computing the similarity measure. Figure 2 presents the region segregation and feature extraction process.

The $R_{i}\;\forall \;r\in R$ is extracted using I and C of an input image. The RS is based on the available $\left(x,y\right)\forall T$ such that r∈R is either a $C_{max}$ or $\;C_{min}\;$extract. Here, either C or I is considered due to the overlapping pixels, and therefore, the regions are identified. This identification is used for specific feature extraction such that $\bar{R}$ matches any of $R_{i}$ (Figure 2). The extracted feature in deep recurrent learning iterations is computed as the number of overlapping features obtained in different regions. In this case, the false rate is mitigated in R due to multiple objects in that raw imagery. Therefore, this false rate for targeted object region selection affects the region at any instance in which the similarity measure is required from the training input matching over the different regions, which is expressed as

(4) $$SM\left(C,\;I\right)=\frac{OV^{f}}{{\left(\frac{C_{Min}}{C_{Max}}-NOV^{f}\right)}^{2}}$$

(5) $$SM_{x,y}=sqrt\left(\frac{\sum_{Np}P^{{image}^{2}}\left(x,y\right)-Np*Mean\left(P^{image}{}_{Np}\left(x,y\right)\right)}{Np-1}\right)$$

(6) $$NOV^{f}=\frac{1}{RS}\sqrt{\frac{1}{r-1}\overset{r}{\underset{x,y=1}{\sum }}{\left(\frac{R-\bar{R}}{Np}\right)}^{2}}$$

Equations (4)–(6) compute the similarity measure based on extracted features and training inputs and outputs in overlapping features $OV^{f}$ and non-overlapping features $NOV^{f}$. Here, the non-overlapping features are distributed, whereas overlapping features are concatenated for identifying the target and the total number of pixels Np in a given image in which the appropriate computation of targeted object region selection is required using a similarity measure in deep recurrent learning iterations. Based on $P^{image}$ and $SM_{x,y}$, the continuous identification of non-overlapping features is expressed as

(7) $$Fx [P^{image},SM_{x,y}]=\sqrt{{ [\frac{SM_{x,y}}{P^{image}}]}_{1}^{2}+{ [\frac{SM_{x,y}}{P^{image}}]}_{2}^{2}+\ldots +{ [\left(1-\frac{\bar{R}}{P^{image}}\right)Np]}_{r}^{2}\;,\;r\in R}$$

Equation (7) estimates the non-overlapping feature from the training inputs until the targeted object region detection is selected due to multiple objects in raw imagery. The selected region pixel size, contrast, and intensity features are analyzed for extraction, relying on the processing time until the similarity verification requires the training input matching in various regions. The above region selection based on the non-overlapping feature is distributed using deep recurrent learning iterations. In this scenario, the populated image must be segregated into regions that must be disseminated in precise processing instances to improve the feature extraction ratio and identify distinguishable regions from the image patch. In addition, the distributed non-overlapping feature is to be instantaneous to perform the pattern matching. Therefore, deep recurrent learning iterations are used for feature extraction and pattern matching. The output of the learning process is to identify and segregate the non-overlapping feature distributed regions through training inputs and previously available data. If the learning process identifies overlapping features in the input image, the distinguishable regions are concatenated for detecting the precisely targeted object. The concatenation for achieving distributed regions and previous information is the best output for targeted object region selection in an image.

### 3.2. Concatenation of Distributed Regions

**Figure 3:**
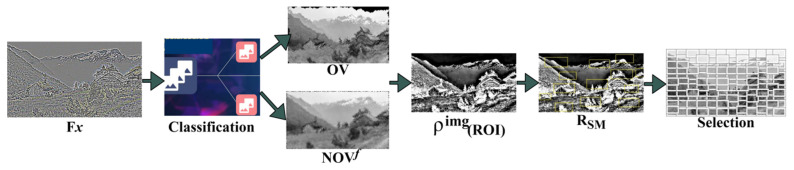
ROI Selection Process.

The similarity measure is computed here and requires the intensity and contrast feature value measured from the raw imagery with 16×16 image regions. The identified overlapping features in distributed and target regions are concatenated with human eye fixations. Multiple sophisticated measures of region-wise feature distribution could be performed in the given image. In densely populated image processing, the region of interest $\left(ROI\right)$ always computes the probability distribution of the image contrast and intensity. The ROI can be estimated with the formula described in the following Equation (8):

(8) $$P^{image}\left(ROI\right)=-\underset{r\in R_{SM}}{\sum }\rho \left(RS\right)\times log\left(\rho \left(RS\right)\right)$$

where $R_{SM}$ is the neighborhood region of the targeted object region identified in the given input image. Figure 3 presents the ROI selection for the Fx processed image.

The Fx input is utilized for classifying OV and $NOV^{f}$ such that $Fx [P^{image},\;SM_{x,y}]$ from which $R_{SM}$ is extracted. This extraction process is required to select precisely $\rho_{P,Q}$ is performed. Therefore, the distribution is performed for concatenation using similarity estimation. Depending on the matching preference, the $SM\left(C,I\right)$ is used for detecting concatenation preference (Figure 3). This represents the probability of maximum possible contrast and intensity over different regions in the analyzed neighborhood. Consider two random variables P and Q, their concatenation can be expressed as in Equations (9) and (10):

(9) $$\rho_{P,Q}=\frac{Concat\left(P,Q\right)}{\Delta_{P}\Delta_{Q}}$$

(10) $$\rho_{P,Q}=\frac{Concat\left(PQ\right)-Concat\left(P\right)Concat\left(Q\right)}{\sqrt{Concat\left(P^{2}\right)-Concat^{2}\left(P\right)}\sqrt{Concat\left(Q^{2}\right)-Concat^{2}\left(Q\right)}}$$

Here, pattern matching is computed at a different location between the overlapping features of 16×16 pixels, and non-overlapping features at distinguishable regions from the given image are computed. It verifies the similarity of training inputs and neighbors for precise target identification. In the distinguishable region concatenation process, the maximum possible region concatenation measures a high special feature in the input image, i.e., low similarity.

### 3.3. Feature Distribution Detection

All features can be extracted except intensity and contrast for training the learning process recurrently; a feature extraction mechanism leads to general extracts in which the overlapped features based on strong peaks at conspicuous locations are identified in a given image while suppressing features that include region selection and peak responses. The false rate is reduced while region-wise feature distribution is carried out using multiple extraction instances with the previously available data; we estimate four statistic values to denote each feature map, such as mean value $M_{V}$, number of maximum possible regions segregate $M_{PR}$ over the pixel, standard deviation $S_{D}$ over the feature map pixels, and the number of peaks $k_{N}$ in the input image feature map. The computation is expressed as in Equations (11)–(13):

(11) $$M_{V}=\frac{1}{x\times y}\underset{T}{\sum }SM_{T}\left(P,Q\right)$$

(12) $$S_{D}=\sqrt{\frac{1}{Np-1}\underset{x,y}{\sum }\left(SM_{T}\left(P,Q\right)-ROI^{2}\right)}$$

(13) $$M_{PR}=mean\left(\sqrt{{\left(x_{P}-x_{Q}\right)}^{2}+{\left(y_{P}-y_{Q}\right)}^{2}}\right)$$

**Figure 4:**
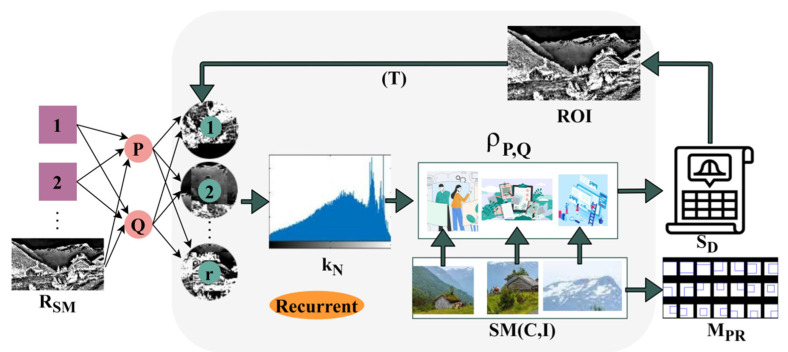
Learning Process.

The deep recurrent learning assessment process from the sequential instances with the first training inputs matching in a different region based on $\;M_{PR}$, $M_{V}$, and $\;S_{D}$ is used for identifying the target object region in the given populated image. Figure 4 presents the learning process.

The $R_{SM}$ is split for P and Q over the varying r∈R such that $k_{N}$ at any variational peaks is identified. The intermediate for $\rho_{P,Q}$ over the $SM\left(C,I\right)\;\forall M_{V}$ is computed; the computation is performed using available r and C,I differences. After the $SM\;\left(C,I\right)$ the OV and $NOV^{f}$ are segregated using C (over I) for $S_{D}$ and $M_{PR}$ detection. This $M_{PR}\in R\;\forall \;t$ is concatenated using $R_{SM}$ for image detection. Contrarily, the $S_{D}$ is reduced for the next ROI such that T is used for the next (consecutive) r ∀P,Q (Figure 4). The region distribution from the training inputs and feature map is used for identifying the overlapping feature and, if this feature can be observed at any region, concatenation is performed and achieves maximum similarity. However, skipping the false rate does not affect the performance of generic target object detection in populated images.

#### Pattern Matching

The pattern-matching estimation is related to the target region selection, except that overlapped feature cooperation across the region is analyzed for precise object detection. The extracted features elaborate the overall populated image data using mean value to denote the pattern matching in that selected region which is identified using multiple extraction instances. The pattern matching can be expressed as

(14) $$\vartheta_{m}=\frac{1}{x\times y}\underset{T}{\sum }{\left(\vartheta_{m}\right)}_{C,I}$$

**Figure 5:**
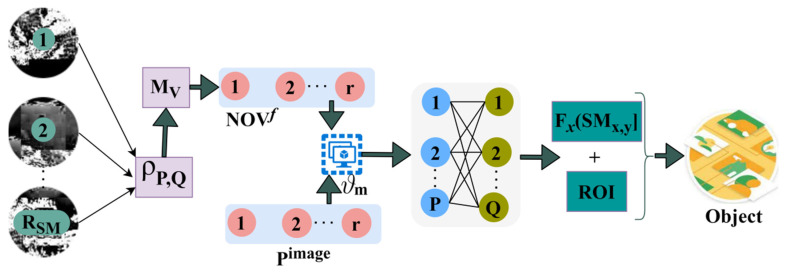
Pattern-Matching Process.

Before performing the region selection and pattern extraction to identify the target in an image, it is necessary to normalize the contrast and intensity feature value. The extracted and normalized feature can be sent to the pattern-matching process for generic target detection. The above representation’s region selection and pattern matching detect a target and satisfy low similarity. The raw imagery’s non-overlapping contrast and intensity features are compared with previously available data and then distributed, for instance. The non-overlapping feature of contrast and intensity jointly produces the output of $Fx [P^{image},SM_{x,y}]$ at its maximum possible concatenation. In this technique, as illustrated in the first and consecutive segregation of the input image into the maximum possible regions, the targeted object location is identified using the training inputs and extracted feature. The pattern-matching process is illustrated in Figure 5.

**Figure 6:**
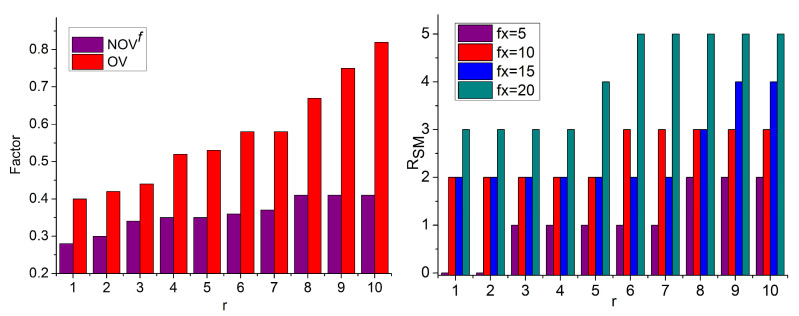
$NOV^{f}$, OV, and$\;R_{SM}$ Analysis.

The pattern matching for the $P^{image}$ and the training inputs are performed using $NOV^{f}$ and OV ∀T. For any single $r\in R_{i}$ identified, the $\vartheta_{m}$ is performed such that $\rho_{P,Q}=maximum$. The possible P,Q combinations are identified $\;\forall F_{x} [SM_{x,y}]$ and $ROI\;\in R_{SM}$ for concatenation. Therefore, the $\vartheta_{m}=maximum$ and $\rho_{P,Q}=maximum$ regions (without OV) are jointly used for detecting the object (Figure 5). In the first instance, the pattern matching is processed for identifying the overlapped and non-overlapped features and maximum peaks in the feature map. Therefore, the maximum similarity feature identification outputs precise target region detection, and hence, the region-wise feature distribution mitigates the false rates and is retained without non-overlapping features, for instance. The false rate-generating features in the images identify distinguishable regions through improved precision wherein the extracted features, such as intensity and contrast, impact the training inputs. The output of the DRL is to identify the overlapping features and mitigates the false rates in region selection and the pattern-matching processes to identify a target. This computation jointly allows generating feature prediction for the instances even if they overlap in the given image for optimum performance of generic target detection. In this manuscript, the possibility of identifying the important difference between the features and regions through DRL and suppressing the non-overlapping features is based on reducing the false rates. Therefore, the minimum false failures and iterations are achieved. Hence, the region selection and pattern matching are consecutively processed using deep recurrent learning iterations to improve precision and identify the region of interest in this image. This generic target detection using similarity verification reduces the processing time and false rate. The extracted feature map and dense embedding features are used for target object detection. The analysis of $NOV^{f}$ and OV and $R_{SM}$ for the varying r is presented in Figure 6.

**Figure 7:**
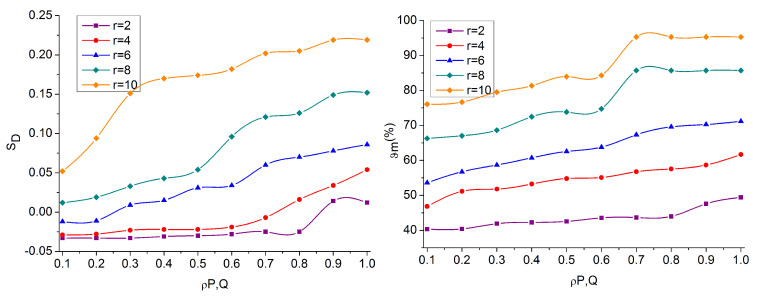
Analysis of $S_{D}$ and $\vartheta_{m}$.

The overlapping and non-overlapping regions are identified using$\;\bar{R}$ from the distribution of $F_{x}\in r$. In the learning process, $k_{N}$ identifies the $\rho_{P,Q}$ for maximization region detection. The $M_{PR}$ from $R_{SM}$ maximizes OV over the identified ROI  and maximizes $C_{max}$ and $C_{min}\;\forall \;F_{x} [P^{image},SM_{x,y}]$. This is required to prevent false rates due to C and I overlapping. Therefore, the need for $R_{SM}$ increases, due to which $S_{D}$ decreases. In this case, the $SM_{xy}$ is validated for identifying any possible input across different $M_{PR}$. Therefore, the presence of a similar region is detected for $\vartheta_{m}$ through recurrent $T\;\forall \;\left(x,y\right)$ (Figure 6). In Figure 7, the analysis of $S_{D}$ and $\vartheta_{m}\left(\%\right)$ is presented.

The proposed technique identifies both negative and positive $S_{D}$ until $0>\rho_{P,Q}$ is observed. This case is modified after $\rho_{P,Q}>0$ or $\rho_{P,Q}=1$; the deviations are suppressed for which r is detected. If the $r\in R_{i}\;\forall \;T$, the $\rho_{P,Q}$ is maximum, then $S_{D}$ reduces; this case is observed in r=8 and 10. This means the regions are high with OV then $NOV^{f}$ and hence $R_{SM}$ is high. Depending on the available $R_{SM}$, the $\vartheta_{m}$ is increased with the available $SM\left(C,I\right)$ and $\bar{R}$. Therefore, the $\bar{R}$ is required to compensate $M_{V}$ without increasing the false rate. This refers to any recurrent iteration $\forall \;S_{D}$ observed.

## 4. Experiments and Results

The performance assessment of the proposed technique is presented in this section using a comparative study. This study analyzes the metrics similarity index, extraction ratio, precision, false rate, and processing time by varying the features and regions. The features are between 2 and 26, and the regions are between 1 and 10. The existing C-FCN [27], CDD-Net [17], and PTAN [19] are augmented with the proposed technique for comparison. The experimental images used in Figure 1, Figure 2, Figure 3, Figure 4 and Figure 5 are extracted from [36,38] with 10,000+ training and testing inputs.

### 4.1. Similarity Index

**Figure 8:**
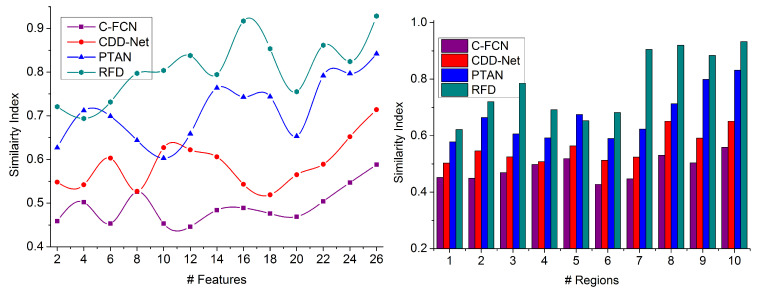
Similarity Index Comparison.

Heterogeneous densely populated images/videos are analyzed to identify the target object region in the feature map through similarity analysis. It refers to skipping the false rate-generating features based on region selection and pattern matching (refer to Figure 8). The intensity and contrast feature achieves a high similarity index required for precise target detection at different time intervals using deep recurrent learning iterations. The region selection and pattern matching are mitigated using multiple extraction instances and the extract feature for $RS\left(C,\;I\right)$ computation. The learning process compares the training inputs and previously available data for gaining similarity measure outputs using contrast and intensity features to augment precision by analyzing 16×16 regions. The continuous image processing identifies distinguishable regions through improved precision for target object detection. This achieves maximum similarity through the overlap feature in a given image/video. Based on image processing, region segregation is used for predicting false rates with improved precision. Therefore, the similarity index is high in this proposed technique.

### 4.2. Extraction Ratio

**Figure 9:**
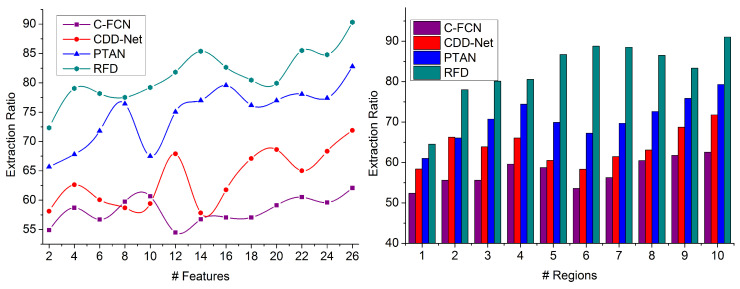
Extraction Ratio Comparison.

This proposed technique achieves a high feature extraction ratio for populated image processing with region segregation; the false rate is mitigated for detecting precise targets from the given image (refer to Figure 9). The region-wise feature distribution is performed for training the DRL recurrently with the contrast and intensity feature over different regions and for objects/people computation. Based on Fx and $\bar{R}$, the similarity is analyzed at various processing time intervals. The proposed technique first segregates the input image into the maximum possible regions with improved precision based on region selection. This false rate is addressed when the target is matched with training inputs for identifying the target. The extraction ratio is estimated over different objects using the previously available data for reducing the similarity measure for gaining high precision in detecting the target object location/region in the feature map. Therefore, $SM\left(C,\;I\right)$ is computed for improving the special feature and the processing time at different regions. Therefore, the similarity verification must satisfy high feature extraction to reduce the processing time. In this proposed technique, image processing is performed to identify the target region and improves precision.

### 4.3. Precision

**Figure 10:**
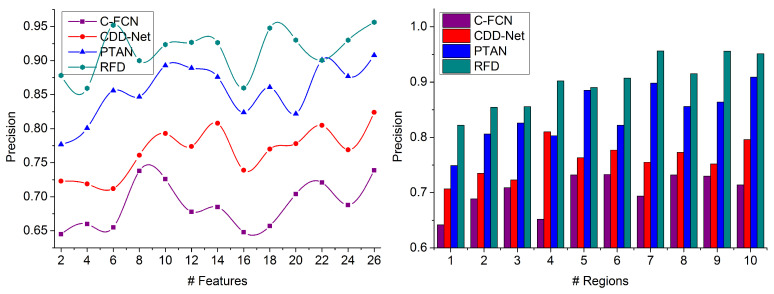
Precision Comparison.

In this proposed technique, the different deep recurrent learning iterations using similarity measures rely on extracted features to more easily detect the target object region in the given populated image due to the presence of multiple objects. Addressing selected regions appropriately and accurately in densely populated images is difficult and region selection and pattern matching are used regarding processing time and training input to reduce the difficulties at different instances. The false rate and multiple objects are identified in the feature map through deep recurrent learning. From the overlapping features instance, the distinguishable regions are concatenated for identifying the target without training input in the feature map based on region segregation through the deep recurrent learning process, preventing false rates. Continuous image processing is performed with similarity feature verification for improving precision. Therefore, the region-wise distribution relies on training inputs for training the learning recurrently. In this proposed technique, the similarity measure is computed for increasing the extraction rate and achieves a lower false rate, as illustrated in Figure 10.

### 4.4. False Rate

**Figure 11:**
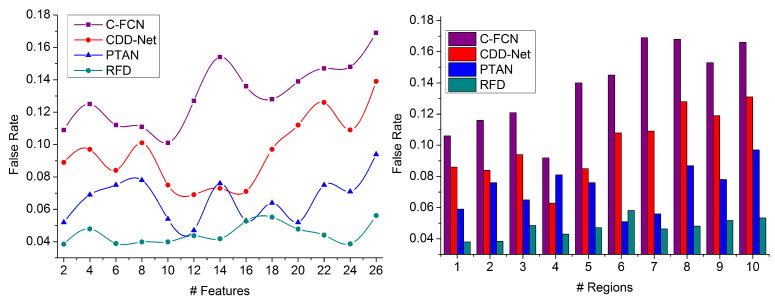
False Rate Comparison.

This proposed RFD technique for detecting a precise target in densely populated images with region selection achieves a lower false rate than other factors, as represented in Figure 11. The distinguishable regions are concatenated for identifying the precise target using a similarity measure. In contrast, the non-overlapping features can be distributed in a densely populated image using deep recurrent learning. Reducing false rate-generating features at different processing time intervals is computed for identifying the target object detection in populated images. From the training inputs, the extracted features and previously available data are matched to detect the generic target to increase the similarity index. The false rate is mitigated in region selection and pattern matching due to multiple objects over different regions. It is difficult to identify the false rate in the feature map in various instances. This technique requires image processing from the training inputs matching in different regions. Thus, the proposed technique estimates three static values for each feature map using multiple extraction instances, and the processing time is less in this analysis.

### 4.5. Processing Time

**Figure 12:**
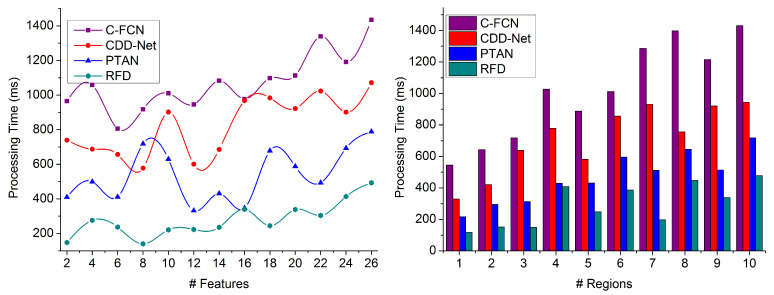
Processing Time Comparison.

The false rate-generating feature is skipped in this proposed technique to identify distinguishable regions. It achieves high processing time for heterogeneous, densely populated image processing (refer to Figure 12). This process improves the precision with previously available data and does not mitigate the false rate compared to the other factors in region-of-interest-based target object detection in the given image. Based on the feature extraction, the overlapping and non-overlapping features are identified through similarity measures for accurate region distribution based on $Fx [P^{image},SM_{x,y}]$ and at its maximum possible concatenation is achieved. In this manner, the maximum similarity leads to appropriate and accurate target object detection in an image through a feature map, and the continuous instance of populated image processing mitigates the false rate during the processing time. This technique reduces processing time and the false rate to maximize precision. These identified false rate-generated features are analyzed and compared with the available data for region selection. Hence, the false rate is mitigated in distinguishable regions for each feature map with less processing time.

### 4.6. Error Probability Ratio

**Figure 13:**
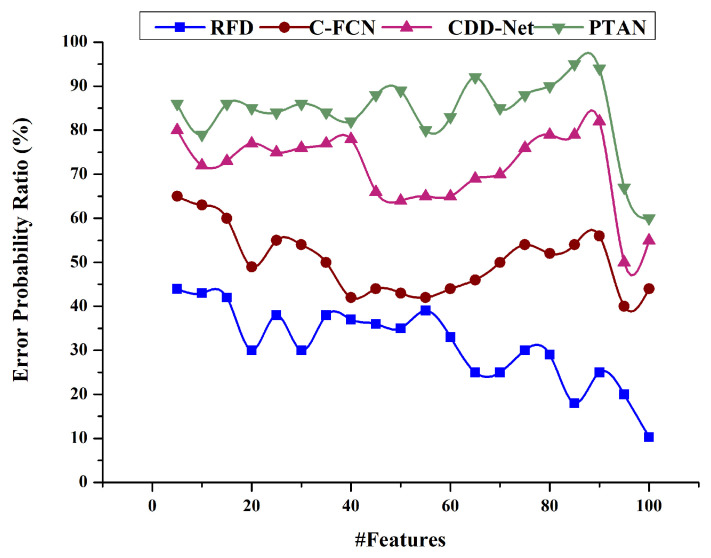
Error Probability Ratio.

This paper defines deep recurrent learning for calculating error probability, frequently utilized with target location estimates. Error probability relates measurement errors to the difference in a calculation calculated from feature measurements. A mathematical basis is provided for the error probability estimate, and deep recurrent learning is shown to be extremely accurate. It varies from the true error probability estimate by less than 1% on average and has a maximum error of 1.5%. As such, it is a beneficial method for evaluating the sensitivity and accuracy of any computed quantity to errored input. The proposed RFD model achieves less error when detecting similar targets. Graphical details of Error Probability Ratio are given in Figure 13.

## 5. Discussion

### Ablation Study

This study presents a series of experiments designed to illustrate the impact of patch size on the model in the RFD. Experiments for single-scale and multi-scale combinations are carried out in this research to cover both the target region and a specific background area to pick patch size. Table 1 shows the patch size for target detection.

In Table 2 and Table 3, the comparative analysis results are summarized. Object-level sensitivity, precision, and accuracy score succeed in achieving reliability. The high-precision and high-sensitivity regions of interest (ROIs) collectively occupy too much of an image on average. Sensitivity analysis, as it is being used in this paper, can be a viable technique for testing the robustness of the DL model to identify how effective the model is when given low-quality images.

## 6. Conclusions

This article introduces a region-focused feature detection technique for identifying specific object targets in densely populated images. The input image is classified based on intensity and contrast for the maximum regions. The regions are distinguished using overlapping and non-overlapping features distributed across different boundaries. For the extracted features within a boundary, the matching for distinguishable features is performed over maximizing the detection precision. The overlapping features with maximum concatenation probability are fused in the alternate, overlapping region for generating the actual image. The fused image is identified from the external inputs across various means and deviations. The entire process is administered using recurrent deep learning to reduce false rates. The new region identification or feature extraction is decided using the learning paradigm for controlled processing time. The proposed RFD improves the similarity index by 10.69%, extraction ratio by 9.04%, and precision by 13.27%. The false rate and processing time are reduced by 7.78% and 9.19%, respectively.

The limitation of the proposed RFD model is the inability to deal with new object classes. The extraction of visual features will be the focus of future research in various environments and weather conditions. These include bright and dim lighting, dense fog, and intense rain. The application of the proposed RFD model includes image classification, surveillance, entertainment, gaming, autonomous vehicles, and scene understanding.

## Figures and Tables

**Table 1:** Patch Size.

Image Type	Patch Size	Number of Training and Testing
Label Image	5, 5, 5, 5	145
Label Image	10, 6, 5, 3	156
Annotated Image	15, 10, 8, 5	278
Annotated Image	20, 16, 15, 10	1610

**Table 2:** Comparative Analysis Results (# Features).

Metrics	C-FCN	CDD-Net	PTAN	RFD
Similarity Index	0.588	0.714	0.842	0.9285
Extraction Ratio	62.09	71.88	82.79	90.326
Precision	0.739	0.824	0.908	0.9564
False Rate	0.169	0.139	0.094	0.0562
Processing Time (ms)	1435.46	1071.21	788.43	492.623

**Findings:** The proposed RFD improves the similarity index by 10.69%, extraction ratio by 9.04%, and precision by 13.27%. The false rate and processing time are reduced by 7.78% and 9.19%, respectively.

**Table 3:** Comparative Analysis Results (# Regions).

Metrics	C-FCN	CDD-Net	PTAN	RFD
Similarity Index	0.559	0.651	0.832	0.9328
Extraction Ratio	62.55	71.79	79.29	91.025
Precision	0.714	0.796	0.909	0.9511
False Rate	0.166	0.131	0.097	0.0534
Processing Time (ms)	1430.83	944.14	717.49	478.172

**Findings:** The proposed RFD improves the similarity index by 12.61%, extraction ratio by 9.91%, and precision by 14.48%. The false rate and processing time are reduced by 7.79% and 8.94%, respectively.

## Data Availability

Not applicable.
